# *Achnanthidium tinea* sp. nov. – a new monoraphid diatom (Bacillariophyceae) species, described on the basis of molecular and morphological approaches

**DOI:** 10.3897/phytokeys.174.60337

**Published:** 2021-03-12

**Authors:** Natalia D. Tseplik, Yevhen I. Maltsev, Anton M. Glushchenko, Irina V. Kuznetsova, Sergei I. Genkal, John Patrick Kociolek, Maxim S. Kulikovskiy

**Affiliations:** 1 K.A. Timiryazev Institute of Plant Physiology RAS, IPP RAS, 35 Botanicheskaya St., Moscow, 127276, Russia; 2 Papanin Institute for Biology of Inland Waters, Russian Academy of Sciences, Yaroslavl, Nekouz, Borok, 152742, Russia; 3 University of Colorado Museum of Natural History, Broadway, Boulder, CO 80302, USA; 4 Department of Ecology and Evolutionary Biology, University of Colorado, Boulder, Colorado, 80309, USA

**Keywords:** *
Achnanthidium
*, Bacillariophyceae, Indonesia, molecular investigations, new species

## Abstract

A new monoraphid diatom species *Achnanthidium tinea* Tseplik, Kulikovskiy, Kociolek & Maltsev, **sp. nov.** is described from Indonesia. The species is described on the basis of molecular and morphological analyses. According to molecular data the new species belongs to the clade that includes strains of *Achnanthidium minutissimum*, *Achnanthidium saprophilum* and *Achnanthidium digitatum*. Morphologically, the new species differs quite significantly from other species of the same genus because of linear-elliptic valves with almost parallel sides and strongly radiate striae and a butterfly-shaped fascia on the raphe valve. The morphology and phylogeny of the new species are discussed, and thoughts on the current state of the taxonomy of the genus *Achnanthidium* are expressed. Our work shows the importance of using molecular data in diatom systematics and also demonstrates the need to investigate rarely studied regions of our planet.

## Introduction

The genus *Achnanthidium* Kützing was first described by Kützing (1844) and for a long time it was considered a subgenus of *Achnanthes* Bory s.l. (Cleve 1895). Its status as a separate genus was restored by Round et al. (1990) and afterwards Round and Bukhtiyarova (1996) proposed a new diagnosis which significantly narrowed the genus boundaries. The improved diagnosis included such features as small linear-lanceolate to elliptic-lanceolate valves, radiate uniseriate striae, external distal raphe ends that are straight or curved to one side and sternum that widens in the center of the valve. Currently, two morphological groups are distinguished within the genus: the *Achnanthidium minutissimum* (Kützing) Czarnecki species complex has straight external distal raphe ends, while the *Achnanthidium pyrenaicum* (Hustedt) Kobayashi species complex has external distal raphe ends that are distinctly curved in one direction (Kobayashi 1997). A third group, previously recognized for *A. exiguum* (Grunow) Czarnecki and its relatives (Karthick et al. 2017), has been established as a separate genus, *Gogorevia* Kulikovskiy, Glushchenko, Maltsev & Kociolek (Kulikovskiy et al. 2020c).

Recent studies include descriptions of many new species belonging to this genus (Rimet et al. 2010; Kulikovskiy et al. 2011; Novais et al. 2011; Krahn et al. 2018; Yu et al. 2019, etc.), as well as studies of type materials of known species using light and scanning electron microscopy (Hlúbiková et al. 2011; Van de Vijver et al. 2011). The studies of type materials primarily concern large species complexes and their main aim is to define separate species more clearly.

Taxonomy within the genus *Achnanthidium* is a rather complicated issue. Species boundaries are often not clear enough due to the fact that morphological features alone may not be sufficient to unequivocally identify species, and because values of quantitative features often overlap in similar species, further complicating their separation (Kulikovskiy et al. 2016a; 2020b; Jahn et al. 2017; Tseplik et al. 2020). These problems require extensive molecular research, both while describing new species and while studying species already known to science. At present, the *Achnanthidium* genus includes about two hundred species (Kociolek et al. 2020b). Molecular data is available for very few taxa, and correct identification of the representatives of this genus based only on morphological features is often difficult (Kulikovskiy et al. 2014, 2016a; Andreeva et al. 2016; Maltsev and Kulikovskiy 2017; Maltsev et al. 2018, 2019).

*Achnanthidium* species are widely distributed in various freshwater habitats around the world and can be important indicators of environmental conditions (Ponader and Potapova 2007). However, many regions remain poorly studied and the probability of finding new species is quite high, like in Lake Baikal (Kulikovskiy et al. 2011, 2012, 2013, 2015, 2016b, c, 2020b) or Southeast Asia (Kulikovskiy et al. 2018; Liu et al. 2018; Glushchenko et al. 2016, 2017, 2018, 2019, 2020; Kezlya et al. 2020). Hustedt (1937a–c, 1938a, b; 1939; 1942) first documented freshwater diatoms from Indonesia, and of the nearly 800 taxa reported, 315 of them (ca. over 40%) were new to science. Still, Indonesia is a country not only with a high level of endemism in many groups of living organisms, but also taxa still to be discovered or reinterpreted (Hamsher et al. 2014; Kapustin et al. 2017, 2019, 2020; Kociolek et al. 2018; Kulikovskiy et al. 2019b, 2020a; Rybak et al. 2019). Of the 39 monoraphid diatoms reported in the genera *Cocconeis* Ehrenberg and *Achnanthes* Bory by Hustedt (1937a–c, 1938a, b; 1939) alone, 10 (26%) were described as new. There have been no modern taxonomic studies of *Achnanthidium* in Indonesia. The purpose of the present report is to provide light and scanning electron microscopic observations, as well as DNA sequence data, in support of the description of a new *Achnanthidium* species from Sulawesi, Indonesia.

## Materials and methods

### Sample collection

The sample used in the present report was collected from Indonesia by I.I. Ivanov on 22.09.2010, and designated I227 from the Sulawesi Island, Temple Lake, periphyton, scraping from macrophytes, t=26.5 °C, pH=8.7, conductivity=277 μS cm^-1^, 04°06.923'N, 119°58.613'E.

### Culturing

Monoclonal strains were established by micropipetting single cells under an inverted microscope. Non-axenic unialgal cultures were maintained in WS liquid medium (Andersen 2005) for one month. The strain investigated here was designated Ind296.

### Preparation of slides and microscopic observation

The sample and the monoclonal culture were treated with 10% hydrochloric acid to remove carbonates and washed several times with deionized water for 12 hours. Afterwards, the samples were boiled in concentrated hydrogen peroxide (≈37%) to dissolve organic matter. After decanting and refilling up to 100 ml with deionized water, the suspension was spread on to coverslips and left to dry at room temperature. Permanent diatom preparations were mounted in Naphrax (refraction index =1.73). Light microscopic (LM) observations were performed with a Zeiss Axio Scope A1 microscope equipped with an oil immersion objective (×100, n.a. 1.4, differential interference contrast) and Axiocam Erc 5s camera (Zeiss). Valve ultrastructure was examined using a JSM-6510LV scanning electron microscope (IBIW, Institute for Biology of Inland Waters RAS, Borok, Russia).

For scanning electron microscopy (SEM), parts of the suspensions were fixed on aluminum stubs after air-drying. The stubs were sputter-coated with 50 nm Au in an Eiko IB 3. Sample and slides are deposited in the collection of MHA, Main Botanical Garden Russian Academy of Science, Moscow, Russia. The type slide was designated 04133.

All images acquired from the slides were processed using Adobe Photoshop CC (19.0). Length and breadth of the valves were measured on the LM images, and striae and areolae density was measured on the SEM images. The numbers given in brackets in the description are means with standard deviations.

### Molecular investigations

Total DNA of monoclonal cultures was extracted using InstaGene Matrix according to the manufacturer’s protocol. A fragment of 18S rDNA (382 bp, including V4 domain) was amplified using primers D512for and D978rev following Zimmermann et al. (2011). Amplification of the 18S rDNA fragment was carried out using the premade mix ScreenMix (Evrogen, Russia) for the polymerase chain reaction (PCR). The conditions of amplification for 18S rDNA fragment were: an initial denaturation of 5 min at 95 °C, followed by 35 cycles at 94 °C for denaturation (30 s), 52 °C for annealing (30 s) and 72 °C for extension (50 s), and a final extension of 10 min at 72 °C.

The resulting amplicons were visualized by horizontal agarose gel electrophoresis (1.5%), colored with SYBR Safe (Life Technologies, United States). Purification of DNA fragments was performed with the ExoSAP-IT kit (Affimetrix, USA) according to the manufacturer’s protocol. 18S rDNA fragment was decoded from two sides using forward and reverse PCR primers and the Big Dye system (Applied Biosystems, USA), followed by electrophoresis using a Genetic Analyzer 3500 sequencer (Applied Biosystems).

Editing and assembling of the consensus sequences were carried out by comparing the direct and reverse chromatograms using the Ridom TraceEdit program (ver. 1.1.0) and Mega7 (Kumar et al. 2016). Newly determined sequence and DNA fragments from 151 other diatoms, which were downloaded from GenBank (taxa and Accession Numbers are given in the Suppl. material 1), were included in the alignments. Three centric diatom species were chosen as the outgroups. The nucleotide sequences of the 18S rDNA gene were aligned separately using the Mafft v7 software and the E-INS-i model (Katoh and Toh 2010). The resulting alignment had lengths of 404 characters.

The dataset was analyzed using the Bayesian inference (BI) method implemented in Beast ver. 1.10.1. (Drummond and Rambaut 2007) to construct phylogeny. For each of the alignment partitions, the most appropriate substitution model was estimated using the Bayesian information criterion (BIC) as implemented in jModelTest 2.1.10 (Darriba et al. 2012). This BIC-based model selection procedure selected TIM1+I+G model, shape parameter α = 0.4210 and a proportion of invariable sites (pinvar) = 0.3400. We used the GTR model of nucleotide substitution instead of TIM1, given that it was the best matching model available for the Bayesian inference method. A Yule process tree prior was used as a speciation model. The analysis ran for 15 million generations with chain sampling every 1000 generations. The parameters-estimated convergence, effective sample size (ESS) and burn-in period were checked using the software Tracer ver. 1.7.1. (Drummond and Rambaut 2007). The initial 25% of the trees were removed, the rest retained to reconstruct a final phylogeny. The phylogenetic tree and posterior probabilities of its branching were obtained on the basis of the remaining trees, having stable estimates of the parameter models of nucleotide substitutions and likelihood. Maximum Likelihood (ML) analysis was performed using the program RAxML (Stamatakis et al. 2008). The nonparametric bootstrap analysis with 1000 replicates was used. The statistical support values were visualized in FigTree ver. 1.4.4 and Adobe Photoshop CC (19.0).

## Results

### Achnanthidium tinea

Taxon classificationPlantaeCocconeidalesAchnanthidiaceae

Tseplik, Kulikovskiy, Kociolek & Maltsev sp. nov.

4E1EA69A-DF2C-5644-B985-D251F6AA213C

Figs 1 , 2 , 3

#### Holotype.

**Figure 1.**
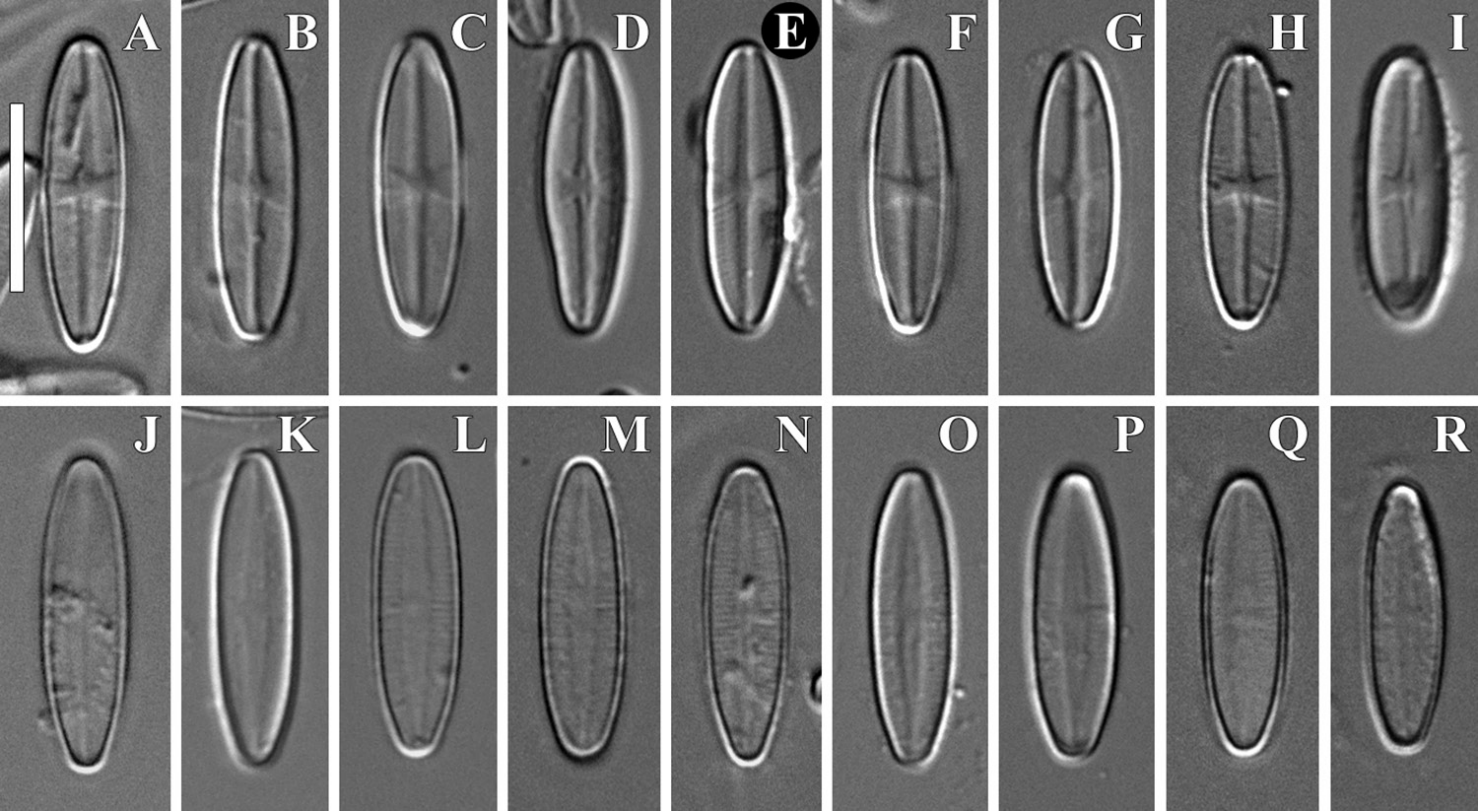
**A–R***Achnanthidium tinea* (Tseplik, Kulikovskiy, Kociolek & Maltsev), sp. nov. LM, DIC, size diminution series. Slide no 04133. Holotype (**E**). Scale bar: 10 μm.

Slide no 04133 in collection of MHA, Main Botanical Garden Russian Academy of Science, Moscow, Russia, represented here by Fig. 1E.

#### Reference strain.

Sample Ind296, isolated in sample I227.

#### Type locality.

Indonesia. Sulawesi Island, Temple Lake, periphyton, 04°06.923'N, 119°58.613'E, 5 m. elev., *leg.* I.I. Ivanov, *22.09.2010*.

#### Description.

***LM*** (Fig. 1A–R). Frustules rectangular in girdle view, raphe valve very slightly concave. Valves linear-elliptic with gradually narrowing ends. Length 14.7–17.5 µm (16.2 ± 0.9; n=17), breadth 4.0–5.0 µm (4.5 ± 0.3; n=17). The raphe valve possesses a straight filiform raphe, which lies in a narrow linear axial area. The central area is represented by a symmetrical butterfly-shaped fascia that reaches the valve margins on both sides. Striae on raphe valve strongly radiate, curved. The rapheless valve possesses a narrow lanceolate axial area. Central area absent, on some valves somewhat shorter striae in the center are present. Striae parallel in the center on the valve, slightly radiate near the valve ends.

**Figure 2.**
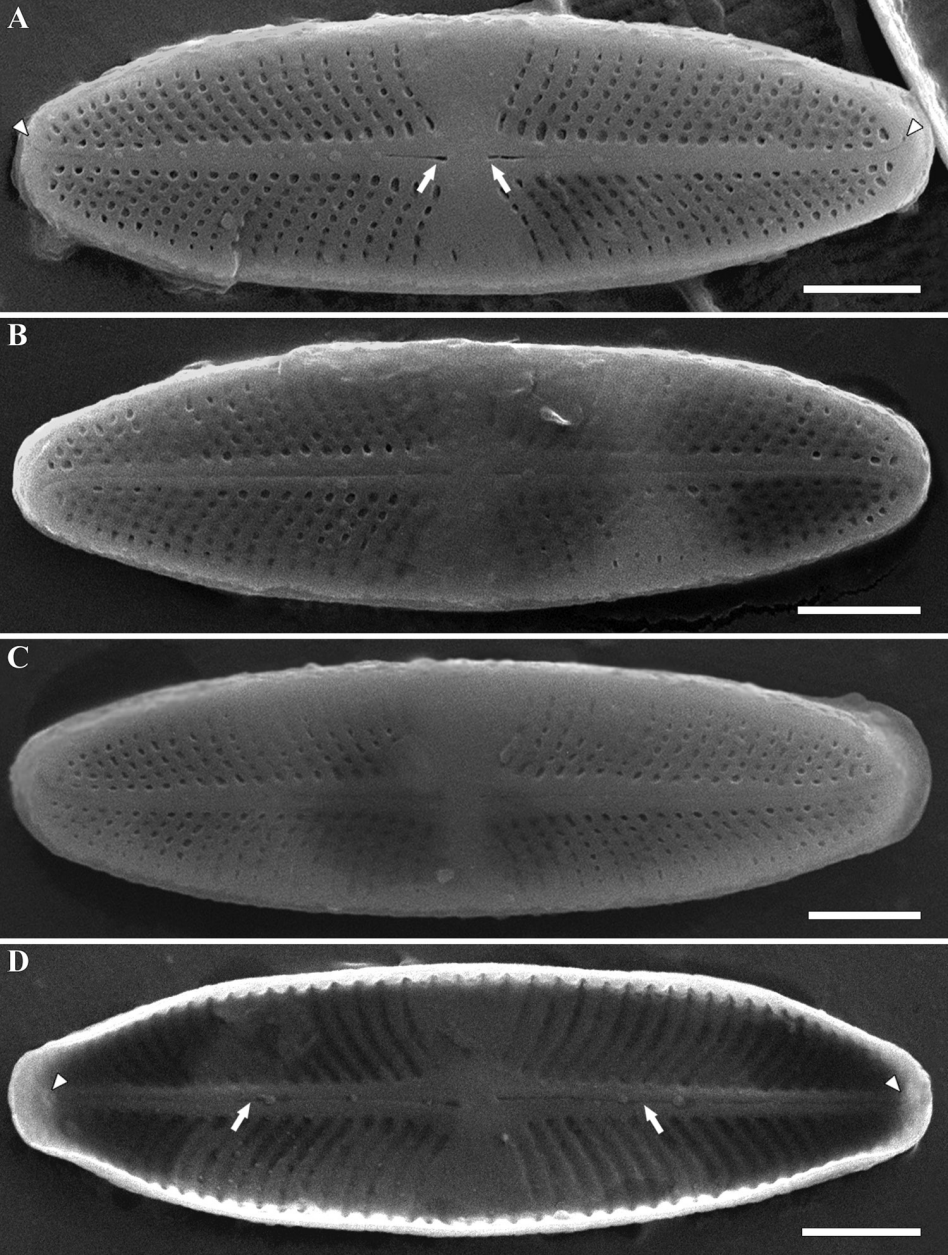
**A–D***Achnanthidium tinea* (Tseplik, Kulikovskiy, Kociolek & Maltsev), sp. nov. SEM. Sample no 04133. Raphe valves **A–C** external views **D** internal view **A** white arrows shows the central raphe ends. White arrowheads shows the distal raphe ends **D** white arrows shows the central raphe ends. White arrowheads shows the helictoglossae. Scale bars: 2 μm.

**Figure 3.**
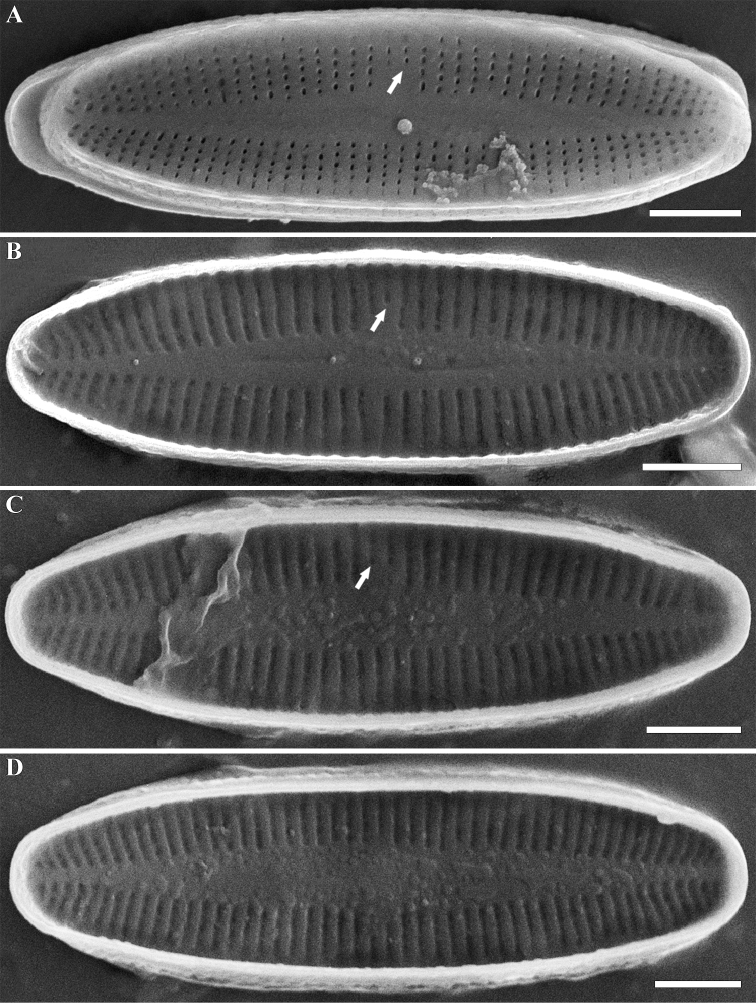
**A–D***Achnanthidium tinea* (Tseplik, Kulikovskiy, Kociolek & Maltsev), sp. nov. SEM. Sample no 04133. Rapheless valves. **A** external view **B–D** internal views **A–C** white arrows shows the short striae. Scale bars: 2 μm.

***SEM, external view*** (Figs 2A–C, 3A). Central raphe ends are straight and drop-shaped (Fig. 2A, white arrows). Distal raphe ends curve strongly to one side of the valve (Fig. 2A, white arrowheads). Striae on the raphe valve 30–35 in 10 µm (32.5 ± 2.5 in 10 µm; n=4). Areolae elliptical or rounded in shape, approximately 40 in 10 µm. Striae on the rapheless valve 30–33 in 10 µm (31 ± 1.2 in 10 µm; n=4). Areolae small, also rounded or elliptical, approximately 50 in 10 µm. Shorter striae in the center are clearly visible in SEM; this often occurs only on one side of the valve (Fig. 3A, white arrow).

***SEM, internal view*** (Figs 2D, 3B–D). Central raphe ends are simple and straight (Fig. 2D, white arrows). Distal raphe ends terminate in helictoglossae (Fig. 2D, white arrowheads). Shorter striae in the center are clearly visible in SEM, this often occurs only on one side of the valve (Fig. 3B, C, white arrows).

#### Etymology.

Epithet refers to the butterfly-like shape of the fascia on the raphe valve of the new species; *tinea* meaning moth in Latin.

#### Distribution.

As yet known only from type locality.

**Figure 4.**
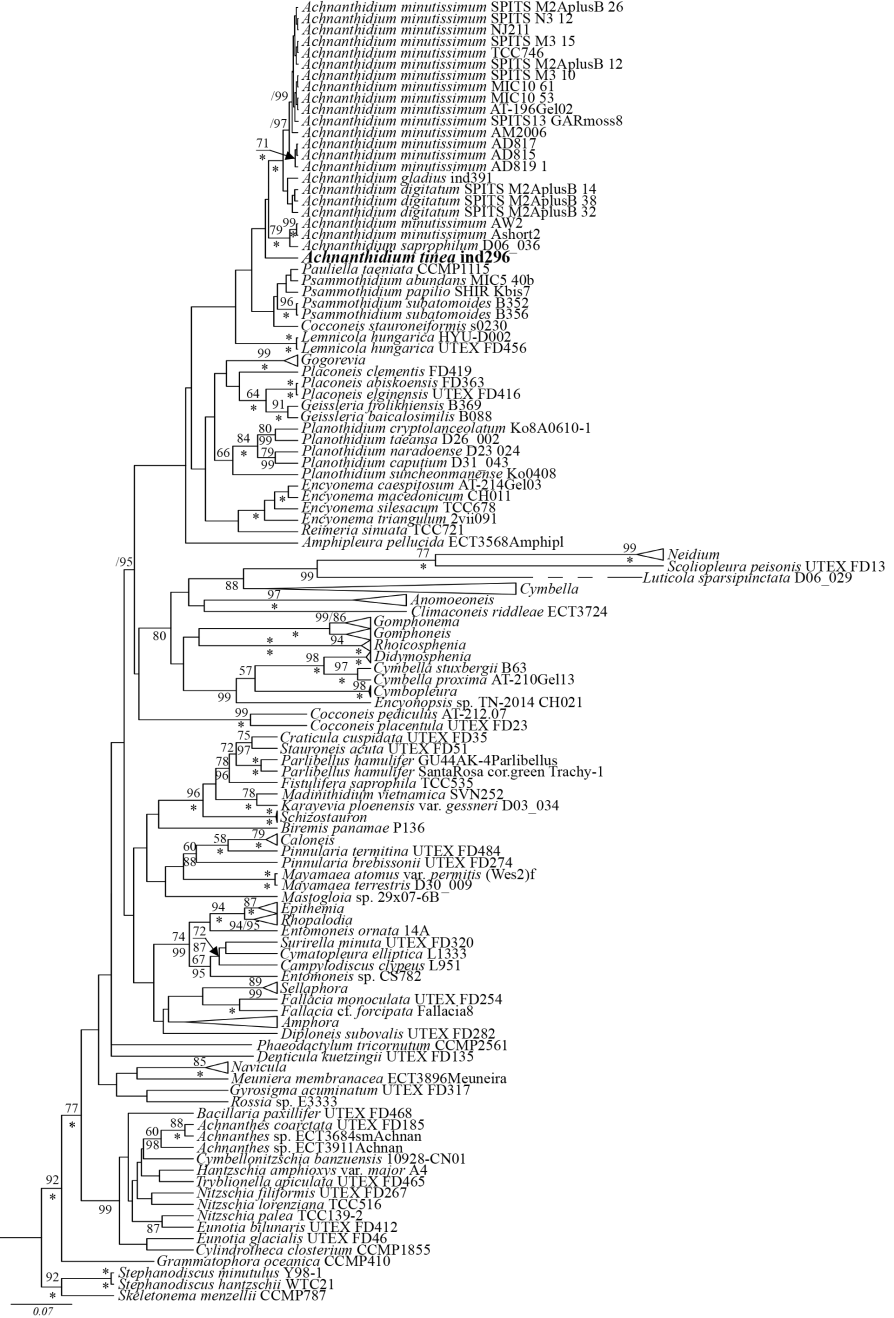
Bayesian tree of *Achnanthidium tinea* (Tseplik, Kulikovskiy, Kociolek & Maltsev), sp. nov. (indicated in bold) constructed from a concatenated alignment of 152 partial 18S rDNA sequences of 404 characters. Values above the horizontal lines are bootstrap support from RAxML analyses (<50 are not shown); values below the horizontal lines and to the right of the slash mark are Bayesian posterior probabilities (<90 are not shown). All sequences have strain numbers (if available). Species of centric diatoms were used as an outgroup. * is 100% statistical support.

Molecular data (Fig. 4)

Our new species belongs to the large clade with monoraphid diatoms and sister clade with gomphocymbelloid diatoms. Strain *A. tinea* sp. nov. combined (BI 100; ML 100) with two strains of *A. minutissimum* AW2 and Ashort2 and *A. saprophilum* D06-036. 15 other strains of *A. minutissimum* combined to form a sister branch together with three strains of *A. digitatum* and *A. gladius* Tseplik et al. Other monoraphid taxa from genera *Pauliella*, *Psammothidium*, *Planothidium*, *Cocconeis*, *Lemnicola* and *Gogorevia* spp. formed sister clades to the branch containing these *Achnanthidium* taxa in the molecular tree.

## Discussion

In terms of the data from both morphology and molecular sequence data, the new species *A. tinea* sp. nov. belongs to the genus *Achnanthidium*. Morphological features present in *A. tinea* and characteristic for this genus include: linear-elliptical valve shape, sternum that widens near the center of the valve and external distal raphe endings that are curved to one side. The last feature allows us to attribute the new species to the *A. pyrenaicum* species complex.

We compared *A. tinea* sp. nov. with other representatives of the genus *Achnanthidium*. *A. tinea* sp. nov. possesses a rather unusual combination of features for the genus: linear-elliptic valves with parallel sides and narrowed ends and a pronounced butterfly-shaped fascia on the raphe valve. After carrying out the morphological comparison, we identified several species most similar in morphology to *A. tinea* sp. nov. In terms of valve shape, the species most similar to the new species is *Achnanthidium deflexum* (Reimer) Kingston (Potapova and Ponader 2004), but it can be quite easily distinguished from *A. tinea* sp. nov. by the absence of the central area and by parallel and more widely spaced striae on the raphe valve (20–22 in 10 μm in *A. deflexum*, 30–35 in 10 μm in *A. tinea* sp. nov.). Another species similar to *A. tinea* sp. nov. in terms of valve shape is *Achnanthidium dolomiticum* Cantonati & Lange-Bertalot (Cantonati and Lange-Bertalot 2006). It differs from the new species by having more widely rounded valve ends and the central area represented by a narrow rectangular fascia. Under the scanning electron microscope, it is also possible to observe straight external distal raphe ends in *A. dolomiticum*, while in *A. tinea* sp. nov. they are curved. *Achnanthidium delmontii* Pérès, Le Cohu & Barthès (Pérès et al. 2012) also resembles *A. tinea* in terms of valve shape and, like *A. tinea* sp. nov., belongs to the *A. pyrenaicum* species complex. But *A. delmontii* has a narrower rectangular fascia and more widely-spaced, weakly radiate striae on both valves (raphe valves: 30–35 in 10 µm in *A. tinea* sp. nov., 20–26 in 10 µm in *A. delmontii*; rapheless valves: 30–33 in 10 µm in *A. tinea* sp. nov., 18–22 in 10 µm in *A. delmontii*). Two other species that somewhat resemble *A. tinea* sp. nov. were studied by Morales et al. (2011), namely *Achnanthidium cadimae* Morales, Fernández & Ector and *Achnanthidium peruvianum* Morales & Ector. *A. cadimae* can be differentiated from our new species by its narrowly elliptic valves that are smaller than *A. tinea* sp. nov. (10–13 μm versus 14.7–17.5 µm), its asymmetrical fascia, and the axial area on its rapheless valve that is very narrow and almost linear versus a somewhat broader lanceolate one in *A. tinea* sp. nov. *A. peruvianum* also has a smaller fascia and a narrower axial area on its rapheless valve than *A. tinea* sp. nov., and its valve ends are more broadly rounded. A final species that is similar in valve shape is *Achnanthes tropica* Hustedt, illustrated with line drawings by Hustedt (1937b, Plate XIII, figs 28–32) and described from Java (Hustedt 1937b, p. 200). This species also has fine striae (reported as “zart” by Hustedt and described as 26–30/10 µm), but coarser than in *Achnanthidium tinea*. While no SEM work has yet been done on Hustedt’s species, the light microscope images of this taxon published by Simonsen (1987, plate 326, figs 20–28) suggest this species might be better placed in the genus *Nupela* Vyverman & Compére.

In general, due to the above-mentioned unusual combination of features possessed by the new species, its similarity with other representatives of the genus is mostly quite superficial, and *A. tinea* sp. nov. is easily distinguishable from other species even in light microscopy.

On the phylogenetic tree, the strain of *A. tinea* sp. nov. forms a separate branch within a clade that includes other species of *Achnanthidium* and other monoraphid diatoms (e.g. *Gogorevia*, *Psammothidium*, *Planothidium*, *Pauliella*, *Cocconeis*, *Lemnicola*) and the Cymbellales. The group was referred to as the Monoplacatae by Mereschkowsky (1902) and has been recovered in previous phylogenetic analyses (e.g. Thomas et al. 2016). Within this large group, *A. tinea* belongs to a large clade comprised of strains of several *Achnanthidium* species, including *A. minutissimum*, *Achnanthidium digitatum* Pinseel, Vanormelingen, Hamilton & Van de Vijver, *Achnanthidium saprophilum* (Kobayashi & Mayama) Round & Bukhtiyarova and *Achnanthidium gladius* Tseplik, Kulikovskiy, Glushchenko & Genkal. As discussed above, morphologically, none of these species is similar to *A. tinea* sp. nov. The clade including this species is sister to another clade that comprises strains of other monoraphid genera, namely *Pauliella* Round & Basson, *Psammothidium* Bukhtiyarova & Round and *Cocconeis* Ehrenberg.

Our understanding of the phylogenetic relationships of the monoraphid diatoms continues to yield fascinating new insights at the levels of genus and species (e.g. Round and Basson 1997; Moser et al. 1998; Witkowski et al. 2000; Krammer and Lange-Bertalot 2004; Riaux-Gobin et al. 2012), and a richer understanding of the evolution of the monoraphid condition (Kociolek et al. 2019). The description of new species within genera such as *Achnanthidium* is quite an important area of research for the taxonomy of this genus, but also shows how the genus might be understood for ecological analyses (Potapova and Hamilton 2007). An integrated molecular and morphological approach to species-level identification and understanding phylogenetic relationships of those taxa will provide a more complete picture of the taxonomy of the genus, allow for the construction of a natural classification, and facilitate further research.

## Supplementary Material

XML Treatment for Achnanthidium tinea

## Supplementary materials

Supplementary material 1 Taxa and DNA sequence data used in phylogenetic analysis Data type species data https://binary.pensoft.net/file/519682This dataset is made available under the Open Database License (http://opendatacommons.org/licenses/odbl/1.0/). The Open Database License (ODbL) is a license agreement intended to allow users to freely share, modify, and use this Dataset while maintaining this same freedom for others, provided that the original source and author(s) are credited.Natalia D. Tseplik, Yevhen I. Maltsev, Anton M. Glushchenko, Irina V. Kuznetsova, Sergei I. Genkal, John Patrick Kociolek, Maxim S. Kulikovskiy
