# Supplementary material for: Achnanthidium tinea sp. nov. – a new monoraphid diatom (Bacillariophyceae) species, described on the basis of molecular and morphological approaches
Source: PhytoKeys. 2021 Mar 12;174:147–63. doi: 10.3897/phytokeys.174.60337 (PMC7979678; doi:10.3897/phytokeys.174.60337)
Supplement: Supplementary material 1 — Taxa and DNA sequence data used in phylogenetic analysis [file phytokeys-174-147-s001.docx]

Supplementary table 1. Taxa and DNA sequence data used in phylogenetic analysis

| taxa | source | the GenBank accession no. for 18S rDNA | taxa | source | the GenBank accession no. for 18S rDNA |
| --- | --- | --- | --- | --- | --- |
|  |  |  |  |  |  |
| *Achnanthes coarctata* (Brébisson ex W.Smith) Grunow | UTEX FD185 | HQ912594 | *Gogorevia ovalis* Kulikovskiy, Glushchenko, Maltsev & Kociolek | VN324 | MT193498 |
| *Achnanthes* sp. | ECT3684smAchnan | KC309476 | *Gogorevia ovalis* | VN352 | MT193499 |
| *Achnanthes* sp*.* | ECT3911Achnan | KC309475 | *Gogorevia ovalis* | VN362 | MT193500 |
| *Achnanthidium digitatum* Pinseel, Vanormelingen, Hamilton & Van de Vijver | SPITS M2AplusB 32 | KX946582 | *Gogorevia renatii* Kulikovskiy, Glushchenko, Maltsev & Kociolek | VN311 | MT193493 |
| *Achnanthidium digitatum* | SPITS M2AplusB 38 | KU565387 | *Gogorevia renatii* | VN342 | MT193494 |
| *Achnanthidium digitatum* | SPITS M2AplusB 14 | KU565386 | *Gogorevia renatii* | VN358 | MT193495 |
| *Achnanthidium gladius* Tseplik, Kulikovskiy, Kociolek & Maltsev | Ind391 | MW025231 | *Gogorevia renatii* | VN386 | MT193497 |
| *Achnanthidium minutissimum* (Kützing) Czarnecki | NJ211 4 | KJ65840 | *Gogorevia renatii* | VN398 | MT193496 |
| *Achnanthidium minutissimum* | SPITS M2AplusB 12 | KU565385 | *Gogorevia uniseriata* (Y. Shi & B.-H. Kim) Kulikovskiy & Kociolek | Ef26 | MT193501 |
| *Achnanthidium minutissimum* | SPITS M3 15 | KU565383 | *Gogorevia uniseriata* | Ef42 | MT193502 |
| *Achnanthidium minutissimum* | TCC746 | KF959663 | *Gogorevia uniseriata* | Ef66 | MT193503 |
| *Achnanthidium minutissimum* | AD819 1 | KJ658400 | *Gogorevia uniseriata* | Ef69 | MT193504 |
| *Achnanthidium minutissimum* | AM2006 | KJ658401 | *Gomphoneis* sp*.* | TN-2014 29vi091A | KJ011649 |
| *Achnanthidium minutissimum* | Ashort2 | KJ658402 | *Gomphonema acuminatum* Ehrenberg | CH042 | KJ011671 |
| *Achnanthidium minutissimum* | AT-196Gel02 | AM502032 | *Gomphonema brebissonii* (Kützing) Grunow | FD373 | KJ011653 |
| *Achnanthidium minutissimum* | AW2 | KJ658403 | *Gomphonema subclavatum* var. *mexicanum* (Grunow) Grunow | FD108 | KJ011666 |
| *Achnanthidium minutissimum* | MIC10 53 | KU565384 | *Gomphonema pumilum* (Grunow) E. Reichardt & Lange-Bertalot | TCC536 | KC736629 |
| *Achnanthidium minutissimum* | MIC10 61 | KU565379 | *Grammatophora oceanica* Ehrenberg | CCMP410 | HQ912634 |
| *Achnanthidium minutissimum* | SPITS M2AplusB 26 | KU565376 | *Gyrosigma acuminatum* (Kützing) Rabenhorst | UTEX FD317 | HQ912598 |
| *Achnanthidium minutissimum* | SPITS M3 10 | KU565377 | *Halamphora coffeaeformis* (C. Agardh) Levkov | UTEX FD75 | HQ912602 |
| *Achnanthidium minutissimum* | SPITS N3 12 | KU565380 | *Hantzschia amphioxys* var. *major* (Ehrenberg) Grunow | A4 | HQ912404 |
| *Achnanthidium minutissimum* | SPITS13 GARmoss8 | KU565378 | *Karayevia ploenensis* var*. gessneri* (Hustedt) Bukhtiyarova | D03_034 | KM084870 |
| *Achnanthidium saprophilum* (H. Kobayashi & Mayama) Round & Bukhtiyarova | D06_036 | KM084866 | *Lemnicola hungarica* (Grunow) Round & Basson | UTEX FD456 | HQ912626 |
| *Achnanthidium minutissimum* | AD817 | KJ658399 | *Lemnicola hungarica* | HYU-D002 | KY354248 |
| *Achnanthidium minutissimum* | AD815 | KJ658398 | *Lemnicola uniseriata* Y. Shi & B.-H.Kim | HYU-D001e | KY354249 |
| ***Achnanthidium tinea* Tseplik, Kulikovskiy, Kociolek & Maltsev** | **Ind296** | **MW025239** | *Luticola sparsipunctata* Levkov, Metzeltin & A. Pavlov | D06_029 | KM084878 |
| *Amphipleura pellucida* (Kützing) Kützing | ECT3568Amphipl | KC309477 | *Madinithidium vietnamica* Kulikovskiy, Andreeva, Maltsev & Kociolek | SVN252 | MH231749 |
| *Amphora helenensis* M.H. Giffen | SZCZCH704 | KT943649 | *Mastogloia* sp. | 29x07-6B | HQ912632 |
| *Amphora pediculus* (Kützing) Grunow | L1030 | HQ912417 | *Mayamaea atomus* var. *permitis* (Kützing) Lange-Bertalot | (Wes2)f | JN418600 |
| *Anomoeoneis fogedii* Reimer | FD399 | KJ011610 | *Mayamaea terrestris* N. Abarca & R. Jahn | D30_009 | KM084909 |
| *Anomoeoneis sculpta* (Ehrenberg) Cleve | CH239 | KJ011611 | *Meuniera membranacea* (Cleve) P.C. Silva | ECT3896Meuneira | KC309482 |
| *Anomoeoneis sphaerophora* Pfitzer | FD160 | KJ011612 | *Navicula cryptocephala* Kützing | 0UTEX FD109 | HQ912603 |
| *Bacillaria paxillifer* (O.F. Müller) T. Marsson | UTEX FD468 | HQ912627 | *Navicula ramosissima* (C. Agardh) Cleve | C87 | AY485512 |
| *Biremis panamae* Barka, Witkowski & Weisenborn | P136 | KM078661 | *Navicula* sp. | 3VIII07N.martyana | KJ577862 |
| *Caloneis lewisii* R.M. Patrick | UTEX FD54 | HQ912580 | *Neidium bisulcatum* (Lagerstedt) Cleve | UTEX FD417 | HQ912591 |
| *Caloneis silicula* (Ehrenberg) Cleve | Cal 890 TM | JN418593 | *Neidium productum* (W. Smith) Cleve | UTEX FD116 | HQ912582 |
| *Campylodiscus clypeus* (Ehrenberg) Ehrenberg ex Kützing | L951 | HQ912412 | *Nitzschia filiformis* (W. Smith) Van Heurck | UTEX FD267 | HQ912589 |
| *Climaconeis riddleae* A.K.S.K. Prasad | ECT3724 | HQ91264 | *Nitzschia lorenziana* Grunow | TCC516 | KC736637 |
| *Cocconeis pediculus* Ehrenberg | AT-212.07 | AM502010 | *Nitzschia palea* (Kützing) W.Smith | TCC139-2 | KF959653 |
| *Cocconeis placentula* Ehrenberg | UTEX FD23 | HQ912592 | *Parlibellus hamulifer* (Grunow) E.J. Cox | GU44AK-4Parlibellus | KJ577866 |
| *Cocconeis stauroneiformis* H. Okuno | s0230 | AB430614 | *Parlibellus hamulifer* | SantaRosa_cor.green_Trachy-1 | KU179137 |
| *Craticula cuspidata* (Kutzing) D.G. Mann | UTEX FD35 | HQ912581 | *Pauliella taeniata* (Grunow) Round & Basson | CCMP1115 | KJ658408 |
| *Cylindrotheca closterium* (Ehrenberg) Reimann & J.C. Lewin | CCMP1855 | HQ912645 | *Pinnularia brebissonii* (Kützing) Rabenhorst | UTEX FD274 | HQ912604 |
| *Cymbella affinis* Kützing | AT-204Gel02 | AM502009 | *Pinnularia termitina* (Ehrenberg) R.M. Patrick | UTEX FD484 | HQ912601 |
| *Cymbella affinis* | AT-213.04 | AM502018 | *Placoneis abiskoensis* (Hustedt) Lange-Bertalot & Metzeltin | FD363 | KJ011667 |
| *Cymbella aspera* (Ehrenberg) Cleve | AT-210Gel07 | AM502016 | *Placoneis clementis* (Grunow) E.J. Cox | FD419 | KJ011668 |
| *Cymbella lanceolata* (C. Agardh) Kirchner | AT-194Gel07 | AM502026 | *Placoneis elginensis* (W. Gregory) E.J. Cox | UTEX FD416 | HQ912607 |
| *Cymbella proxima* Reimer | AT-210Gel13 | AM502017 | *Planothidium caputium* J. Zimmermann & R. Jahn | D31_043 | KY650801 |
| *Cymatopleura elliptica* (Brébisson) W. Smith | L1333 | HQ912659 | *Planothidium cryptolanceolatum* R. Jahn & N. Abarca | Ko8A0610-1 | KY650803 |
| *Cymbopleura naviculiformis* (Auerswald ex Heiberg) Krammer | AT-221.02 | AM502004 | *Planothidium naradoense* R. Jahn & J. Zimmermann | D23_024 | KY650795 |
| *Cymbopleura naviculiformis* | AT-177.04 | AM501997 | *Planothidium suncheonmanense* R. Jahn & J. Zimmermann | Ko0408 | KY650802 |
| *Cymbella stuxbergii* (Cleve) Cleve | B63 | KJ011627 | *Planothidium taeansa* R. Jahn & N. Abarca | D26_002 | KY650796 |
| *Cymbellonitzschia banzuensis* Stephanek, Hamscher, S. Mayama, Jewson & Kociolek | 10928-CN01 | KT693310 | *Phaeodactylum tricornutum* Bohlin | CCMP2561 | HQ912556 |
| *Denticula kuetzingii* Grunow | UTEX FD135 | HQ912610 | *Psammothidium abundans* (Manguin) Bukhtiyarova & Round | MIC5 40b | KU565381 |
| *Didymosphenia dentata* (Dorogostaisky) Skvortsov & K.I.Meyer | B547 | KJ011635 | *Psammothidium papilio* (D.E. Kellogg, Stuiver, T.B. Kellogg & G.H. Denton) K. Kopalová & B. Van de Vijver | SHIR Kbis7 | KU565382 |
| *Didymosphenia geminata* (Lyngbye) Mart.Schmidt | B40 | KJ011637 | *Psammothidium subatomoides* (Hustedt) Bukhtiyarova & Round | B356 | unpublished |
| *Diploneis subovalis* Cleve | UTEX FD282 | HQ912597 | *Psammothidium subatomoides* | B352 | unpublished |
| *Entomoneis ornata* (Bailey) Reimer | 14A | HQ912411 | *Reimeria sinuata* (Grunow) Kociolek & Stoermer | TCC721 | KT072996 |
| *Entomoneis* sp. | CS782 | HQ912631 | *Rhoicosphenia abbreviata* (C. Agardh) Lange-Bertalot | CH030 | KJ011672 |
| *Encyonema caespitosum* Kützing | AT-214Gel03 | AM502035 | *Rhoicosphenia abbreviata* | CH229 | KJ011673 |
| *Encyonema macedonicum* Z. Levkov, Metzeltin & S. Krstic | CH011 | KJ011638 | *Rhopalodia contorta* Hustedt | L1299 | HQ912406 |
| *Encyonema silesacum* (Bleisch) D.G. Mann | TCC678 | KF959662 | *Rhopalodia gibba* (Ehrenberg) O. Müller | CH155 | HQ912407 |
| *Encyonema triangulum* (Ehrenberg) Kützing | 2vii091 | KJ011645 | *Rossia* sp. | E3333 | EF15196 |
| *Encyonopsis* sp*.* | TN-2014 CH021 | KJ011646 | *Schizostauron* sp. | SZCZP1010 | KT943592 |
| *Epithemia argus* (Ehrenberg) Kützing | CH211 | HQ912408 | *Schizostauron* sp. | SZCZP39 | KT943593 |
| *Epithemia sorex* Kützing | CH148 | HQ912409 | *Scoliopleura peisonis* Grunow | UTEX FD13 | HQ912609 |
| *Epithemia turgida* (Ehrenberg) Kützing | CH154 | HQ912410 | *Sellaphora blackfordensis* D.G. Mann & S. Droop | (Bfp5x8)F1-3 | JN418599 |
| *Eunotia bilunaris* (Ehrenberg) Schaarschmidt | UTEX FD412 | HQ912599 | *Sellaphora capitata* D.G. Mann & S.M. McDonald | BLA10 | EF151971 |
| *Eunotia glacialis* F.Meister | UTEX FD46 | HQ912586 | *Sellaphora pupula* (Kützing) Mereschkovsky | AUS1 | EF151982 |
| *Fallacia* cf. *forcipata* (Greville) Stickle & D.G. Mann | Fallacia8 | EF151960 | *Skeletonema menzellii* (Greville) Cleve | CCMP787 | AY684940 |
| *Fallacia monoculata* (Hustedt) D.G. Mann | UTEX FD254 | HQ912596 | *Stauroneis acuta* W. Smith | UTEX FD51 | HQ912579 |
| *Fistulifera saprophila* (Lange-Bertalot & Bonik) Lange-Bertalot | TCC535 | KF959658 | *Stephanodiscus hantzschii* Grunow | WTC21 | DQ514914 |
| *Geissleria baicalosimilis* M. Kulikovskiy, E. Gusev, S. Andreeva & N. Annenkova | B088 | KJ787113 | *Stephanodiscus minutulus* (Kützing) Cleve & Möller | Y98-1 | DQ514916 |
| *Geissleria frolikhiensis* M. Kulikovskiy, E. Gusev, S. Andreeva & N. Annenkova | B369 | KJ787114 | *Surirella minuta* Brébisson ex Kützing | UTEX FD320 | HQ912658 |
| *Gomphoneis minuta* (Stone) Kociolek & Stoermer | CH053 | KJ011648 | *Tryblionella apiculata* W. Gregory | UTEX FD465 | HQ912600 |
